# Effects of Personal Low-Frequency Stimulation Device on Myalgia: A Randomized Controlled Trial

**DOI:** 10.3390/ijerph19020735

**Published:** 2022-01-10

**Authors:** Yong-Soon Yoon, Myoung-Hwan Ko, Il-Young Cho, Cheol-Su Kim, Johny Bajgai, Hong-Young Jang, Ka-Eun Kim, Kyu-Jae Lee, Mihyun Lee

**Affiliations:** 1 Department of Physical Medicine & Rehabilitation, Presbyterian (Jesus) Medical Center, 365, Seowon-ro, Wansan-gu, Jeonju-si 54987, Korea; gvcdr@daum.net; 2 Department of Physical Medicine and Rehabilitation, Jeonbuk National University Medical School, 567, Baekje-daero, Deokjin-gu, Jeonju-si 54896, Korea; mhko@jbnu.ac.kr; 3 Department of Medical Sciences Convergence Research Center for Medical Sciences, Jeonju University, 303, Cheonjam-ro, Wansan-gu, Jeonju-si 55069, Korea; chirotrust@jj.ac.kr; 4 Department of Environmental Medical Biology, Wonju College of Medicine, Yonsei University, Wonju-si 26426, Korea; cs-kim@yonsei.ac.kr (C.-S.K.); johnybajgai@yonsei.ac.kr (J.B.); 5 Department of Physical Education, Sungkyul University, 53, Seonggyeoldaehak-ro, Manan-gu, Anyang-si 14097, Korea; brighthong0@sungkyul.ac.kr; 6 Department of Alternative Medicine, Graduate School of Health and Welfare, Jeonju University, 303, Cheonjam-ro, Wansan-gu, Jeonju-si 55069, Korea; kecam07@jj.ac.kr

**Keywords:** electrotherapy, myalgia, personal low-frequency stimulation, physical therapy, surface electromyography

## Abstract

Electrotherapy is commonly used for myalgia alleviation. Low-frequency stimulation (LFS) is primarily used for controlling acute and chronic pain and is a non-invasive therapy that can be easily performed with electric stimulation applied on the skin. However, little evidence exists regarding the pain alleviation effects of personal low-frequency stimulation device for home use. Moreover, no studies have compared myalgia alleviation effects between personal low-frequency stimulation (PLS) and physical therapy (PT), which are most commonly used for patients with myalgia in hospitals and clinics. Therefore, we aimed to investigate the pain alleviation effects of PLS in patients with myalgia and compare these effects with those of conventional PT (transcutaneous electrical nerve stimulation + ultrasound). In total, 39 patients with myalgia in the neck, shoulder, back, and waist areas were randomly assigned to the personal low-frequency stimulation group (PLSG: *n* = 20) and physical therapy group (PTG: *n* = 19). Both groups were treated for 3 weeks (20 min per session and 5 sessions per week). Patients were assessed for pain intensity by surface electromyography (sEMG), visual analogue scale (VAS) and a short-form McGill pain questionnaire (SF-MPQ) before and after the intervention period. Our results showed that PLSG showed a tendency of muscle relaxation with a significant decrease in sEMG in the neck (*p* = 0.0425), shoulder (*p* = 0.0425), and back (*p* = 0.0046) areas compared to the control group. However, there was no significant difference in waist area. Additionally, VAS scores significantly decreased between pre- and post-treatment in both PTG (*p* = 0.0098), and PLSG (*p* = 0.0304) groups, but there was no significance difference between the groups. With respect to SF-MPQ, the PLSG showed greater pain alleviation (5.23 ± 0.25) effects than the PTG (6.23 ± 0.25). Accordingly, our results suggest that PLS treatment using a home device might offer positive assistance in pain alleviation for patients with myalgia that is as equally effective as conventional PT treatment. However, further detailed studies are required considering larger samples to fully claim the effectiveness of this device.

## 1. Introduction

Musculoskeletal disorders (MSDs) are defined as conditions that include a wide range of inflammatory and degenerative conditions that adversely affect the tendons, muscles, ligaments and peripheral nerves in the body with subsequent pain and discomfort [1]. MSDs are a very common and costly global public health issue and have a substantial impact on an individual’s quality of life. Among these disorders, persistent pain in the neck–shoulder and back regions due to work-related problems, including myalgia, is one of the common symptoms, particularly in females [2,3,4]. In order to decrease this global public health problem, the use of interventions that demonstrate efficacy for specific outcomes is clearly essential. Recently, increased attention has been paid by therapists to evaluate the efficacy of various conservative therapeutic interventions to manage pain. Towards this, electrotherapy (ET) is a commonly used intervention for alleviation of myalgia through stimulation of muscle and analgesia [5]. Over the past few decades, numerous studies have been conducted on ET as a potent non-pharmacological treatment alternative for the treatment of painful conditions like fibromyalgia in which peripheral nociception and central processing of pain occurs [6,7,8]. In ET, low-frequency stimulation is primarily used for relieving acute and chronic levels of pain. These non-invasive neuro-stimulation devices were invented shortly after the proposal of the gate control theory of pain by Melzack and Wall [9]. Low-frequency transcutaneous stimulation activates large-diameter nerve fibers and releases excitatory neurotransmitters that quickly reach the brain. In contrast, another branch extending from the large-diameter nerve fiber activates interneuronal fibers to release inhibitory neurotransmitters. This, in turn, reduces the activities of secondary transmitter cells in the central nervous system that carry pain signals, which results in the reduction of pain signals that were continuously inputted into the brain [9,10,11].

Advancing knowledge and research on pain mechanism has spurred attempts to develop more effective strategies to treat pain at home by the innovation of personal medical care appliances. A personal low-frequency stimulation (PLS) device (CGM MBD-1201; CERAGEM Inc., Cheonan-si, Korea) was developed and approved as a medical care device for the purpose of pain reduction by the Korean Food and Drug Administration. This medical device might have the potential to alleviate myalgia through treatment of low-frequency stimulation (LFS) on the site of pain and can be operated easily in home settings. For treatment, a gel pad is placed onto the surface of the skin to apply pulsatile electric stimulation, and the frequency, intensity, and sustained time of stimulation can be adjusted. On the other hand, transcutaneous electrical nerve stimulation (TENS) devices are generally utilized in hospitals for the purpose of pain control. TENS has been proven to be effective for pain symptoms associated with conditions like back pain, osteoarthritis, pancreatic cancer, and fibromyalgia [6,12,13,14,15]. However, until now, very little evidence on the pain alleviation effects of PLS in home settings has been recorded. Moreover, there are no studies to date that have compared myalgia alleviation effects between PLS and conventional physical therapy (PT) that is most commonly applied to patients with myalgia in hospitals and clinics. Therefore, in this study, we aimed to investigate the pain alleviation effects of PLS in patients with myalgia and compare these effects with those of conventional PT.

## 2. Materials and Methods

### 2.1. Participants

Thirty-nine female adults aged between 18 and 70 years old were recruited at the department of rehabilitation medicine, Presbyterian Medical Center, located in Jeonju, Korea. These participants visited the center due to complaints of prolonged muscular pain. The participants were diagnosed as having myalgia by experienced clinicians at the department of rehabilitation medicine, Presbyterian Medical Center on the basis of muscle and general physical examination, and prolonged muscular pain for at least 3 months. Participants experienced muscle pain in at least one or more sites of the neck, shoulder, back, and waist areas. This study selected female patients who satisfied the inclusion criteria based on the screening test and submitted a signed informed consent form (ICF) after receiving a detailed explanation about the clinical trial. Patients were excluded in this study if they had any diseases of the organs, such as liver disease, cancer, rheumatic disease, thyroid disease, skin disorders, or hypersensitivity; those with skin desensitization; those who had undergone drug administration or medical device applications in another clinical trial within 30 days from the start of the present study; and those whom the investigator determined to be ineligible to participate in the clinical trial (e.g., serious instability, mental illness, minors less than 18 years of age, spinal pain, osteoporosis, and those who could not visit the center for all five sessions per week for 3 weeks). Furthermore, all the selected participants (*n* = 39) were randomly assigned to either the PLS group (PLSG: *n* = 20) or PT group (PTG: *n* = 19).

### 2.2. Experimental Design

**Figure 1:**
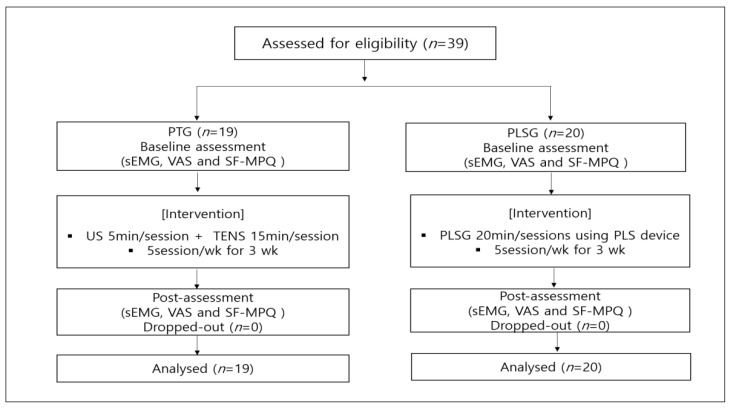
Flowchart of the general experimental design and number of participants in this clinical trial. PLS, personal low-frequency stimulation; PT, physical therapy; sEMG, surface electromyography; SF-MPQ, short-form McGill questionnaire; TENS, transcutaneous electrical nerve stimulation; US, ultrasound; VAS, visual analogue scale.

A randomized controlled parallel design was used in this study. The patients underwent acclimation and screening tests, including medical inquiry, health screening, and physical examination for 7 days before starting the clinical trial. Eligible participants were randomly assigned by a computer program into two groups, (1) PLSG or (2) PTG, and registered in this clinical trial. All participants signed the ICF and underwent measurements for baseline values prior to receiving the designated interventions. All interventions were administered for 3 weeks, and the values were re-measured upon completion of the interventions. The PLSG received the intervention by using a Youridm device (CGM MBD-1201; CERAGEM Inc., Cheonan-si, Korea) at a low-frequency mode for 20 min per session and 5 sessions per week. The PTG underwent a conventional PT consisting of ultrasound (US) (SONICATOR 740, Mettler Electronics, CA, USA) for 5 min and TENS (IN-1000A, YOUNG-IN biotech Co. Ltd., Seoul, Korea) for 15 min, for a total of 20 min per session and 5 sessions per week. Both groups were allowed to use drug therapy (non-steroidal anti-inflammatory drugs and acetaminophen). The present study was approved by the Institutional Review Board of the Presbyterian Medical Center (IRBN. 2019-09-038). The overall experimental design is shown in Figure 1.

### 2.3. Experimental Device (PLS Device)

**Figure 2:**
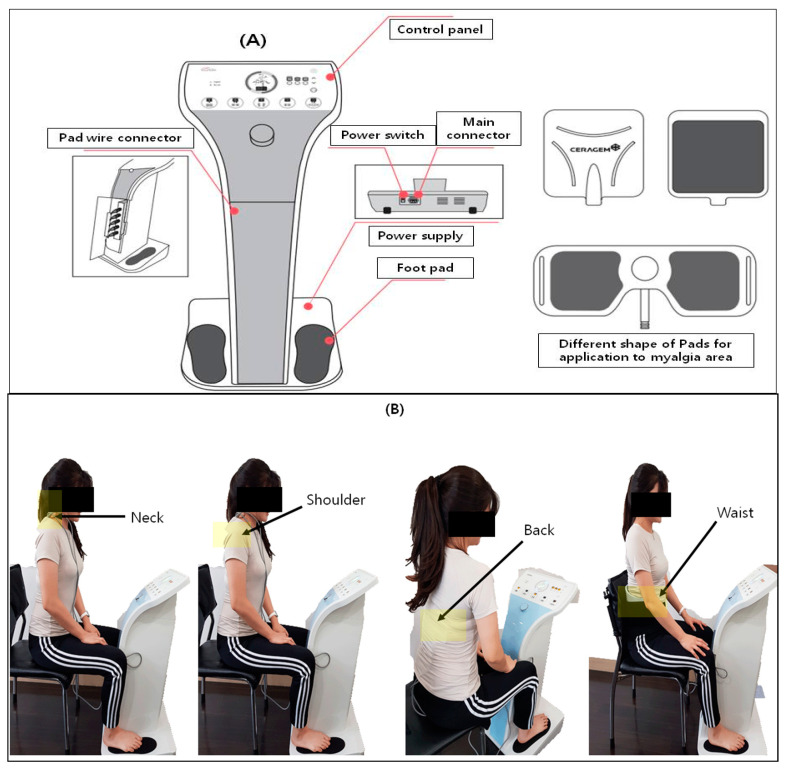
(**A**) Personal low-frequency stimulation device (CGM MBD-1201) and (**B**) clinical application of PLS on four different positions, such as the neck, shoulder, back, and waist.

The PLS device is a personal combinational stimulator (CGM MBD-1201, Youridm, CERAGEM Inc., Cheonan-si, Korea; Figure 2) including the functions of low-frequency stimulation (LFS), ultrasound, and heating. However, in this study, we only used the LFS function of the device to evaluate the myalgia alleviation effects. For the treatment of PLSG, the participant sat on a chair with their feet on each silicon foot pad on which gel was applied to increase electrical conductivity, and also attached the other pad onto the neck, shoulder, back, and waist areas where there was pain. For all the participants, only the most painful area was selected among the neck, shoulder, back, and waist regions even if they had pain in two sites. Treatment was conducted in LFS mode 3 which was set with a frequency of 20–70 Hz ± 10%, a maximum output current of 6.9 mA ± 20%, and a pulse width of 630 µs ± 10%. The total treatment time was 20 min and the interval of frequency switching time was 3 s. For the control treatment, US was set at a frequency of 1.5–1.8 mHz for 5 min, and TENS was set at a frequency of 2–80 Hz, with a pulse width of 700 µs ± 10% and frequency switching time of 1 s for 15 min and applied onto the neck, shoulder, back, or waist areas where there was pain. The total treatment time of the control group was 20 min.

### 2.4. Sample Size

The number of the participants included in this study was determined based on the root mean square (RMS) value of surface electromyography (sEMG). According to the results of Kim et al. [16], the RMS value of the lumbar extensor at rest was 22.46 ± 10.59 before treatment and 11.81 ± 11.06 after treatment, with an average change of 10.65. For the estimation of the number of samples, a continuous independent t-test was used. The treatment effect was calculated based on a power of 80%. A *p*-value of 0.05 was considered statistically significant. Accordingly, 15 participants were required in each group. We estimated a dropout rate of 30%, and thus aimed to allocate 20 participants to each group in the current study.

### 2.5. Outcomes Measures

#### 2.5.1. Surface Electromyography

Muscle activity was assessed using sEMG FREEEMG (BTS Bioengineering Corp, Milan, Italy). The measured values were assessed through signal processing of the RMS, and the measured value used was the average of the left and right values. The device was attached to the skin in four regions (neck, shoulder, back, and waist), and values were measured at rest and during the contraction–relaxation exercise. The measurement method for each area was as follows. (1) At resting position: With the feet spread apart at shoulder width, the knees were slightly bent and both hands were supported on top of the knees. After assuming a comfortable position, the position was maintained for 25 s. (2) Neck area assessment position: After a rest period, the investigator pulled the head of the participant forward as much as possible for 5 s, while the participant maintained the motion of applying force in the opposite direction. (3) Shoulder area assessment position: After a rest period, participants were instructed to sit in the trunk upright posture and contracted their shoulder muscles for 5 s, and were told to hold position for 10 s. (4) Back area assessment position: After a rest, the participant moved to a bed that was set up on the side. While lying in a prone position, the participant applied maximum force to lift the upper body using only the back muscles. To measure the maximum force at this time, the investigator applied an opposing force to the upper body of the participant and maintained it for 5 s. (5) Waist area assessment position: After a rest period, the participant moved to a bed that was set up on the side. While lying in a prone position, the participant applied maximum force to raise the feet without bending the knees by using only the lower back muscles. To measure the maximum force at this time, the investigator applied an opposing force to the lower body of the participant and maintained it for 5 s. For all the participants, only one most painful area was selected among the neck, shoulder, back, and waist regions even if they had pain in two sites.

#### 2.5.2. Subjective Pain

Subjective pain scores were measured using a visual analogue scale (VAS) and short-form McGill questionnaire (SF-MPQ). A VAS is a 10 cm scale with the left end indicating “no pain” and the right end indicating “very severe pain” [17]. VAS is commonly known as a tool to measure for sensitivity and proportions of pain, and is a widely used method for chronic as well as acute pain with good reliability [17]. On a scale numbered from 0 to 10, the participant subjectively marked their pain level.

SF-MPQ is a multi-dimensional questionnaire for measuring pain that consists of 15 items: 11 sensory words and 4 affective words for pain [18]. Using a 4-point scale (0: no pain, 1: weak pain, 2: average pain, and 3: severe pain), the sum score for each of the 15 words was calculated and used in the analysis.

### 2.6. Data Analysis

Descriptive statistics were analyzed to derive the mean and standard deviation by using a SPSS ver. 22.0 program. To test the differences in subjective pain scores (SF-MPQ) between the PLSG and PTG, analysis of covariance (ANCOVA) was performed with pre-test values set as covariates. The effect size was presented as ƞ^2^ (eta) and the results were interpreted based on the ƞ^2^ criteria (large: >0.14, medium: 0.13–0.06, and small: 0.05–0.01). Frequencies of VAS (pre- and post-) distribution were performed to evaluate the subjective pain. To test the differences in sEMG and VAS results, unpaired t tests were performed using the Graph Pad Prism 8.0 software package (Graph Pad, La Jolla, CA, USA). Each participant had different initial VAS. Therefore, we determined the fold change of VAS by the ratio of the changes between the post-VAS value and the pre-VAS value over the pre-VAS value. Differences were considered statistically significant at *p* < 0.05.

## 3. Results

### 3.1. General Characteristics of Participants

A total of 39 female participants enrolled and participated in the study. Of these, 19 participants were randomized to the PTG intervention group and 20 participants were randomized to the PLSG intervention group. The general characteristics of two intervention groups are presented in Table 1. 

### 3.2. Changes in sEMG after PTG and PSLG

**Figure 3:**
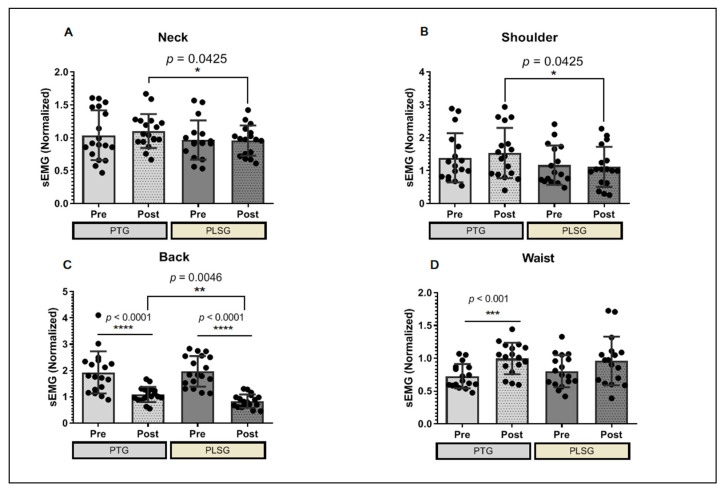
Changes in sEMG values for PTG and PLSG. (**A**) sEMG score at the neck, (**B**) shoulder, (**C**) back, and (**D**) waist areas of participants. Significant differences were considered statistically at * *p* < 0.05, ** *p* < 0.01, *** *p* < 0.001 and **** *p* < 0.0001. PLSG, personal low-frequency stimulation group; PTG, physical therapy group; sEMG, surface electromyography.

The participants were subjected to a relative sEMG analysis to investigate for improvements in sEMG between two pain-reduction therapy techniques. Despite the small number of participants in each group, PLSG had a minimal effect on the neck when compared to PTG, and it has been demonstrated that PLSG application reduces muscle activity significantly more than PTG, which also has a marginal influence on neck muscular discomfort (Figure 3A). The PLSG considerably reduced the sEMG in the shoulder area, indicating that the treatment was similarly effective to PTG in alleviating shoulder discomfort (Figure 3B). The most favorable effects of PLSG were observed in the back muscles (Figure 3C). Both PLSG and PTG significantly reduced the sEMG of the back muscle, resulting in improved pain control. Moreover, the PLSG showed a greater reduction in sEMG, indicating that PLSG is effective in reducing back muscular discomfort. Pain management therapy appears to be less effective around the waist. The sEMG of the waist muscle increased after PTG therapy, indicating that the condition had worsened. On the contrary, this result was not observed in the PLSG (Figure 3D).

### 3.3. Changes in VAS Score after Treatment of PT and PLS

**Figure 4:**
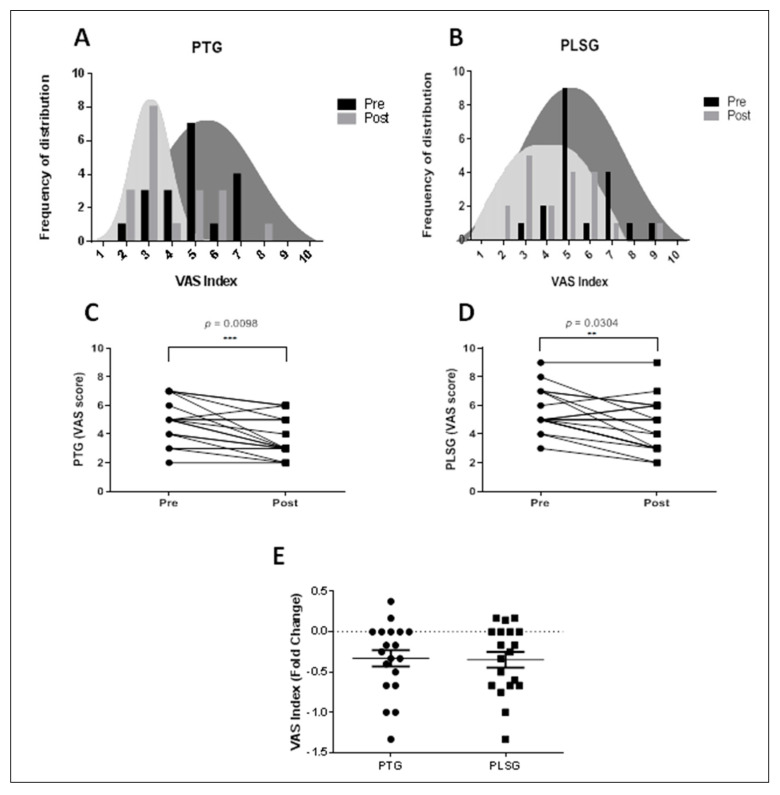
Changes in VAS score for PTG and PLSG pre− and post−treatment. (**A**) Frequency distribution of VAS index for PTG; (**B**) frequency distribution of VAS index for PLSG; (**C**) VAS score for PTG; (**D**) VAS score for PLSG; and (**E**) VAS index expressed in fold changes. Significant differences were considered statistically at ** *p* < 0.01 and *** *p* < 0.001. PLSG, personal low-frequency stimulation group; PTG, physical therapy group; VAS, visual analogue scale.

To examine the effects of PTG and PLSG on perceived pain intensity, VAS was utilized to evaluate pain reported by subjects. The VAS values for both pain treatment regimes decreased, indicating pain abatement (Figure 4). Surprisingly, the frequency of distribution of VAS in the PLSG was higher than in the PTG prior to therapy, implying that some patients in PLSG felt more discomfort than PTG participants. Following PLSG treatment, the VAS score dispersion tends to flatten. When the VAS index is standardized between groups, there are no significant differences in VAS fold change between the PTG and PLSG (Figure 4E), suggesting that both interventions reduced perceived pain levels.

### 3.4. Changes in Subjective Pain Level (SF-MPQ Scores) after Treatment of PT and PLS

Our SF-MPQ scores results (F = 6.558, *p* = 0.015, ƞ^2^ = 0.154) were significantly lower in the PLSG than in the PTG, indicating that pain alleviation was more effective in the PLSG than in the PTG (Table 2).

### 3.5. Safety

To check for safety, all participants were asked questions about adverse effects every time after device application. No members of the PLSG or the PTG complained of any pain or discomfort during the intervention period.

## 4. Discussion

In recent years, a number of studies suggested that an ET field was involved in numerous biologic processes which are of great importance for therapeutic interventions. Particularly, low-frequency fields are known to be a safe and effective therapy and are suggested as a promising adjuvant for treating various musculoskeletal disorders, including pain management [19,20,21]. In this current study, we used PLS therapy for treatment of musculoskeletal pain in older adults. Additionally, we compared our intervention with a combination of US and TENS, which are commonly used interventions for typical myalgia alleviation in PT, based on the validation of its pain reduction effect by numerous researchers [22,23]. Our results suggest that PLS treatment using a home device might offer positive assistance in pain alleviation that is as equally effective as conventional PT interventions in the neck, shoulder, and back regions. Additionally, no side-effects and withdrawals were recorded during the 3 week intervention period.

Accordingly, our results showed significant differences between the PLSG and PTG with respect to changes in sEMG values. sEMG, which was used as a tool for muscle activation in the present study, has been highly correlated with pain experienced by patients in the back and neck regions [24,25], while the patterns of change in sEMG signals in the para-lumbar muscle due to muscle spasms have also been reported to be useful as objective assessment data when there is chronic myalgia in the waist area [26]. In this study, sEMG values for the neck, shoulder, and back areas were found to be significantly reduced in PLSG as compared to PTG. However, in the waist area, the PLSG and PTG showed similar values in sEMG values. Previously conducted studies have reported that sEMG values were found to be elevated in chronic back pain [27], whereas our results showed PLSG to significantly decrease muscle activity and more so than PTG, which indicates that 3 weeks of PLS therapy is effective for relaxing the muscle activity in patients with back pain problems more than PTG. In addition, a study conducted by Naderhand and colleagues reported that patients with neck disability had higher sEMG scores on muscle activity [28]. Similarly, our findings demonstrated that sEMG values were higher in the neck area of patients, whereas with PLS treatment, the scores decreased for patients, more so than PTG. In addition, subjective pain scores showed a significant decrease between pre- and post-treatment with both therapies. Our results showed significant alleviation of pain effects between the pre- and post-tests based on VAS scores in each group, but there was no difference between the two groups. However, the PLSG showed higher alleviation effects than the PTG based on SF-MPQ scores. These findings suggest that PLS could be helpful in alleviating myalgia. Low-frequency stimulation is a safe and inexpensive non-pharmacological therapeutic method generally used to control pain. While there is no direct comparison of myalgia alleviation effects of PLS due to the lack of published study results, TENS used in hospitals has been reported to show positive effects on chronic pain in various randomized clinical trials and, thus, the discussion is based on such results.

A study tested the validity of TENS in 29 patients with peripheral neuropathy [29] and found that pain scores decreased after 6 weeks of intervention, reporting that such an intervention could be useful for patients who do not want additional drug therapy following chemotherapy. To test the effect of TENS on patients with pancreatic cancer [15], a comparison was made between a control group that received analgesics as the intervention and an experimental group that received TENS without analgesics. The results showed an immediate decrease in pain after the intervention in the control group, followed by a gradually increasing tendency after 1, 2, and 3 h. The experimental group, despite not using any analgesics, showed a pattern of excellent pain control, and since such an effect was sustained for 3 weeks after the intervention without any increase in analgesics used, the intervention was proposed as an alternative pain treatment modality for patients with pancreatic cancer. Additionally, a study compared the changes in the pain level in patients with osteoarthritis of the knee based on a single intervention by using high-frequency, low-frequency, and sham TENS groups. The results showed that the pressure pain threshold in the anterior tibial muscle increased in the low-frequency and high-frequency TENS groups [14]. A study assessed the effectiveness of TENS devices in 28 patients with fibromyalgia [30] and found that the groups that received TENS showed a greater effect on indicators related to pain, job performance, fatigue, stiffness, anxiety, and depression than the group that did not. In addition, a recent study on the TENS effect on back pain and neuropathic pain reported that 15 sessions of TENS therapy significantly improved the VAS score pre-and post-treatment and was considered as an effective and safe method of therapy for pain control [31]. These findings are consistent with our study, suggesting that a personal low-frequency electro-stimulation device could be recommended as a home health-care product for alleviating conditions like myalgia. The present study has some notable limitations. First, the study period was limited to 3 weeks of treatment. Therefore, future studies are needed with an extended clinical trial period to determine the exact mechanism and to examine the changing effects at different time points. Second, the study was conducted in a single center and the sample size was small. Third, the drug intake doses could not be recorded during the study period. Therefore, further studies with a larger sample number with a comparison of the treatment according to gender to verify the benefits of this treatment for a longer period is necessary.

## 5. Conclusions

In our study, we found a significant effect of PLS treatment on sEMG and SF-MPQ scores as compared to conventional PT. Specifically, our results demonstrated that sEMG muscle activity for the neck, shoulder, and back areas were significantly reduced in PLSG. In addition, both PTG and PLSG brought about a significant reduction in the VAS score after treatment. Therefore, non-pharmacological therapeutic measures such as PLS can be an effective device for the treatment of myalgia. However, more detailed studies using larger samples are required to fully claim these effects.

## Figures and Tables

**Table 1:** Characteristics of the participants.

Variable	PTG (*n* = 19)	PLSG (*n* = 20)
Age (years)	53.00 ± 14.82	58.95 ± 5.39
BMI (kg/m^2^)	23.18 ± 2.79	25.26 ± 4.61
VAS (score)	4.84 ± 1.50	5.50 ± 1.57
Duration of pain (months)	68.05 ± 68.01	102.10 ± 151.10

Abbreviation. PLSG: personal low-frequency stimulation group, PTG: physical therapy group, BMI: body mass index, VAS: visual analogue scale. Data are express in ± SD (standard deviation).

**Table 2:** Analysis of covariates of changes in the subjective pain level for each group.

	Group	Pre-Test M ± SD	Post-Test M ± SD	Adjusted Post-Test M ± SE	F-Value	ƞ^2^
SF-MPQ	PTG (*n* = 19)	6.26 ± 2.31	5.11 ± 2.26	6.23 ± 0.25	6.558 *	0.154
	PLSG (*n* = 20)	8.55 ± 3.56	6.35 ± 3.67	5.23 ± 0.25		

Abbreviation. PLSG: personal low-frequency stimulation group, PTG: physical therapy group, SF-MPQ: short-form McGill questionnaire, M: mean, SD: standard deviation, SE: standard error,* *p* < 0.05 values show statistically significance.

## Data Availability

All the data are included within the article.
